# AI-driven prediction of treatment outcome in aligner therapy: A pilot study

**DOI:** 10.6026/973206300213404

**Published:** 2025-09-30

**Authors:** Leela Venkata Soujanya Nallamilli, Faisal Mohiuddin Ansari, Preetham Ravuri, Ajay K. Kubavat, Udayini Monica M., Bhagyalakshmi Avinash, Rahul Tiwari, Anil Managutti

**Affiliations:** 1 Department of Orthodontics and Dentofacial Orthopaedics, Narsinhbhai Patel Dental College and Hospital, Sankalchand Patel University, Visnagar, Gujarat, India; 2 Consultant Orthodontist, Al Meswaak Dental Clinic, Riyadh, Saudi Arabia; 3 Department of Orthodontics, Kamineni Institute of Dental Sciences, Narketpally, Telangana, India; 4 Department of Orthodontics, JSS Dental College and Hospital, JSS Academy of Higher Education and Research, Mysore, Karnataka, India; 5 Department of Oral and Maxillofacial Surgery, Narsinhbhai Patel Dental College and Hospital, Sankalchand Patel University, Visnagar, Gujarat, India

**Keywords:** Artificial Intelligence, clear aligner therapy, treatment outcome, predictive modelling, orthodontics

## Abstract

Clear aligner therapy is popular, but predicting whether patients will need refinement remains difficult. In this pilot study, 30 adults undergoing aligner therapy were assessed with an AI model trained on 2000 historical cases to forecast outcomes. The model predicted treatment completion without refinement with 70% accuracy, showing 75% sensitivity and 66.7% specificity. These findings indicate that AI can moderately predict refinement needs and may support personalized treatment planning in orthodontics.

## Background:

Clear aligner therapy (CAT) has become increasingly popular due to its aesthetic and comfortable nature [1]. However, predicting whether a patient will require treatment refinement remains a clinical challenge [2, 3]. Recent advances in artificial intelligence (AI) and machine learning (ML) offer new tools for predictive analytics in orthodontics [4]. In particular, logistic regression with regularization, XG Boost and support vector classification has shown utility in medical prediction tasks [5, 6]. A large retrospective cohort study of nearly 10,000 CAT patients demonstrated moderate predictive performance-with AUC values around 0.67 and precision-recall metrics indicating fair calibration-using these ML algorithms to assess refinement risk [7, 8]. AI models identified key predictors such as patient compliance, interproximal reduction and planned tooth movements [9]. These findings suggest AI could support clinical decision-making by stratifying patients according to refinement risk and tailoring treatment planning accordingly [10]. Therefore, it is of interest to study of AI-driven prediction of treatment outcome in aligner therapy.

## Materials and Methods:

This pilot study employed a prospective observational design to assess an AI model predicting CAT treatment outcomes. Thirty adult patients (age range 18-50 years) undergoing aligner therapy at a university orthodontic clinic was consecutively enrolled. Inclusion criteria included permanent dentition and no systemic or periodontal conditions affecting tooth movement. Pre-treatment data collected comprised age, gender, initial malocclusion severity (classified as mild, moderate, or severe), planned use of interproximal reduction (IPR), attachments, number of aligner steps and patient compliance measured via self-report and device sensors. An AI model employing logistic regression with L1 regularization was trained using anonymized historical data (n ≈ 2000) from the same clinic. The model was designed to predict completion of therapy without the need for refinement. Predictor variables mirrored those collected prospectively. The dataset was split into training (80%) and validation (20%) sets. Model performance metrics included accuracy, sensitivity, specificity and the area under the receiver operating characteristic curve (AUC). For the prospective sample, model predictions regarding need for refinement were recorded and compared against actual outcomes at treatment completion. Statistical analysis used descriptive statistics to summarize demographic and clinical features. Model performance in the pilot cohort was quantified via percent accuracy, sensitivity (true positive rate), specificity (true negative rate) and confusion matrix.

## Results:

The pilot sample included 30 patients: mean age 27 years (SD ± 6), 60% female and malocclusion severity defined as mild (40%), moderate (40%) and severe (20%). IPR was planned in 50% of cases, with attachments used in 70%. Mean number of aligner steps was 25 (range 18-32); compliance averaged 90% wear time (Table 1 - see PDF). The AI model predicted no-refinement in 18 patients and refinement needed in 12. Actual outcomes: 20 completed therapy without refinement; 10 required a second-phase refinement. Accuracy was 70% (21/30 correct predictions); sensitivity (correctly predicting no refinement) was 75% (15/20); specificity (correctly predicting refinement) was 66.7% (6/10) (Table 2 - see PDF).

## Discussion:

This pilot study demonstrates that an AI-based predictive model using routinely collected clinical variables can forecast the need for refinement in CAT with a moderate accuracy of 70 %. In line with prior large-scale findings (AUC ≈ 0.67, similar precision metrics), our findings affirm AI's potential in stratifying patients by risk of treatment refinement [11, 12]. Moreover, our pilot performance-sensitivity 75 % and specificity 66.7 %-aligns with real-world predictive accuracy levels reported in AI applications for orthodontic outcomes [13, 14]. The primary strength of our pilot lies in its real-world application using a prospective cohort within routine clinical workflow. Variables such as compliance, IPR and treatment complexity, which have been identified as influential predictors in ML models, were feasibly collected and contributed meaningfully to risk stratification [15, 16]. Our model's moderate sensitivity suggests it may reliably identify patients more likely to complete treatment without refinement, enabling clinicians to allocate resources or intensify monitoring more effectively. However, several limitations warrant discussion. First, the sample size of 30 is small; results should be interpreted cautiously. A larger sample would enhance statistical power and facilitate better calibration. Second, the model was trained on retrospective clinic data, which may differ in distribution from the prospective cohort, potentially affecting generalizability [17]. Third, specificity was lower, indicating the model was less accurate at identifying those requiring refinement; false negatives in this context may lead to unanticipated treatment delays.

The clinical implications are promising. If refined and validated, such AI tools could be integrated into treatment planning software to highlight high-risk cases for proactive adjustment or patient counseling. Early identification of likely refinement may reduce treatment time and enhance patient satisfaction by setting realistic expectations. Comparison with broader AI applications in orthodontics underscores the value of predictive modeling. AI systems have achieved up to 73 % prediction accuracy and significant reductions in treatment time and appointment frequency [18]. Remote monitoring AI tools have even achieved 82 % accuracy in predicting aligner derailment, reducing unscheduled visits by 28 % [19, 20]. These results collectively affirm AI's transformative potential in CAT. Future research should focus on expanding sample sizes, integrating multi-center data and exploring advanced models (e.g., XGBoost, ensemble approaches) that may increase predictive power. Calibration strategies, such as SHAP analysis, could enhance interpretability and clinician trust. Additionally, evaluating patient-centered outcomes-such as satisfaction and perceived efficiency-alongside predictive performance will be critical.

## Conclusion:

AI can predict refinement need in aligner therapy with ~70 % accuracy, supporting its potential role in personalized treatment planning. Further validation in larger cohorts is warranted.
